# Natural product derivative Gossypolone inhibits Musashi family of RNA-binding proteins

**DOI:** 10.1186/s12885-018-4704-z

**Published:** 2018-08-10

**Authors:** Lan Lan, Hao Liu, Amber R. Smith, Carl Appelman, Jia Yu, Sarah Larsen, Rebecca T. Marquez, Xiaoqing Wu, Frank Y. Liu, Philip Gao, Ragul Gowthaman, John Karanicolas, Roberto N. De Guzman, Steven Rogers, Jeffrey Aubé, Kristi L. Neufeld, Liang Xu

**Affiliations:** 1 Departments of Molecular Biosciences, University of Kansas, 4002 Haworth Hall, 1200 Sunnyside Avenue, Lawrence, KS 66045-7534 USA; 2 Protein Production Group, NIH COBRE in Protein Structure and Function, Lawrence, USA; 3 Center for Computational Biology, University of Kansas, Lawrence, Kansas USA; 4 School of Chemistry and Chemical Engineering, Southeast University, Nanjing, China; 5 Program in Molecular Therapeutics, Fox Chase Cancer Center, Philadelphia, PA USA; 6 Eshelman School of Pharmacy, University of North Carolina, Chapel Hill, NC USA; 7 Department of Radiation Oncology, University of Kansas Cancer Center, Kansas City, Kansas USA; 8 Current address: School of Pharmacy, Southwest Medical University, Luzhou City, China

**Keywords:** Gossypolone, Musashi, RNA-binding protein, Colon cancer, Liposomes

## Abstract

**Background:**

The Musashi (MSI) family of RNA-binding proteins is best known for the role in post-transcriptional regulation of target mRNAs. Elevated MSI1 levels in a variety of human cancer are associated with up-regulation of Notch/Wnt signaling. MSI1 binds to and negatively regulates translation of *Numb* and *APC* (adenomatous polyposis coli), negative regulators of Notch and Wnt signaling respectively.

**Methods:**

Previously, we have shown that the natural product (−)-gossypol as the first known small molecule inhibitor of MSI1 that down-regulates Notch/Wnt signaling and inhibits tumor xenograft growth in vivo. Using a fluorescence polarization (FP) competition assay, we identified gossypolone (Gn) with a > 20-fold increase in Ki value compared to (−)-gossypol. We validated Gn binding to MSI1 using surface plasmon resonance, nuclear magnetic resonance, and cellular thermal shift assay, and tested the effects of Gn on colon cancer cells and colon cancer DLD-1 xenografts in nude mice.

**Results:**

In colon cancer cells, Gn reduced Notch/Wnt signaling and induced apoptosis. Compared to (−)-gossypol, the same concentration of Gn is less active in all the cell assays tested. To increase Gn bioavailability, we used PEGylated liposomes in our in vivo studies. Gn-lip via tail vein injection inhibited the growth of human colon cancer DLD-1 xenografts in nude mice, as compared to the untreated control (*P* < 0.01, *n* = 10).

**Conclusion:**

Our data suggest that PEGylation improved the bioavailability of Gn as well as achieved tumor-targeted delivery and controlled release of Gn, which enhanced its overall biocompatibility and drug efficacy in vivo. This provides proof of concept for the development of Gn-lip as a molecular therapy for colon cancer with MSI1/MSI2 overexpression.

**Electronic supplementary material:**

The online version of this article (10.1186/s12885-018-4704-z) contains supplementary material, which is available to authorized users.

## Background

The expression of the RNA-binding protein Musashi-1 (MSI1) is elevated in a variety of human cancers, including glioblastoma, breast, colon and lung cancers [1–10], with higher levels corresponding to poor prognosis [3–5, 10–12]. Msi1 was first identified in *Drosophila* where it plays a role in neural development and asymmetric cell division in the adult sensory organ [13]. Subsequently, Msi1 homologs were identified in other species, with higher levels in stem and undifferentiated cells [1, 2, 14–17]. Musashi-1 typically plays a role in post-transcriptional regulation of target mRNAs [18–22]. Up-regulation of MSI1 in cancers appears to associate with elevated Notch/Wnt signaling, as MSI1 targets *Numb* [22, 23] and *APC* (adenomatous polyposis coli) [19] are negative regulators of Notch and Wnt signaling, respectively [24, 25]. *CDKN1A* (*P21)*, a negative regulator of cell cycle progression, is also a direct MSI1 target [21]. In all three cases, MSI1 blocks target mRNA translation. Knocking down MSI1 using siRNA [3], miRNA [26] and a small molecule inhibitor [27] led to decreased xenograft tumor growth. Taken together, these results point to MSI1 as a potential therapeutic target.

Our previous study identified (−)-gossypol as a small molecule inhibitor of MSI1 that reduced cancer cell proliferation and xenograft growth [27]. More recent screening in our lab using an FP assay revealed more potent and/or specific inhibitors of MSI1. One inhibitor with a Ki of 12 ± 2 nM against full length MSI1 was gossypolone (Gn), it had a higher affinity than (−)-gossypol (Ki = 476 ± 273 nM) [27]. Gn also showed similar affinity towards Musashi-2 (MSI2) in FP assay (Ki = 7.0 ± 0.3 nM against full length MSI2). MS12 is another Musashi family member that plays both redundant and independent roles as MSI1 in neural stem cells [28, 29]. In cancer, MSI2 expression is elevated in hematologic malignancies [30–36], colorectal adenocarcinomas [37], lung [38], pancreatic cancers [39–41], and glioblastoma [42]. MSI1 and MSI2 share sequence and structure similarity, especially their N-terminal RNA recognition motifs (RRMs). The residues that recognize r(GUAGU) are highly conserved between MSI1 and MSI2 [43]. Thus, Gn can potentially be used as a MSI1/2 dual inhibitor.

Gn is a major metabolite of gossypol [44], and is oxidized in the liver by P450 enzyme [45]. Gn shares similar biological activities as gossypol [46–52], including as an inhibitor of Bcl-2 family with a Ki of 0.28 μM toward Bcl-xL [49]. However, in colon cancer cell assays, the same concentration of Gn was less potent than (−)-gossypol [27].

To address this problem, we introduce a new liposome-based Gn nanocarrier. Liposomes have long been used as nanocarriers for targeted cancer therapy and have demonstrated biocompatibility and controlled drug release in previous studies [53–56]. Particularly, compared with unmodified liposomes, some PEGylated liposomes were reported to be less entrapped by reticuloendothelial cells and lead to enhanced drug delivery to solid tumors in vivo [57–59]. In the present study, PEGylated liposomes were used to improve the bioavailability of Gn as well as to achieve tumor-targeted delivery and controlled release of Gn, which enhances its overall biocompatibility and drug efficacy in vivo.

## Methods

### Cell culture and reagents

Human colon cancer cell lines HCT-116, HCT-116 β/W and DLD-1, are as described by Lan et al. [27] and tested for mycoplasma contamination [60] before use.

Gossypolone (Gn) was prepared as previously described [61]. (3, 4-Dimethoxyphenyl)methanimine gossypol (MP-Gr) was synthesized from gossypol [27]. The Gn and MP-Gr powder were dissolved in DMSO at 20 mM as stock solutions. L-α-phosphatidylcholine (EPC) and 1,2-distearoyl-sn-glycero-3-phosphoethanolamine-N-[methoxy(polyethylene glycol)-2000] (PEG-DSPE) were purchased from Avati Polar Lipids, Inc. (Alabama, USA) . DiR (1,1′-dioctadecyl-3,3,3′,3′-tetramethylindotricarbocyanine iodide) was purchased from Invitrogen (Carlsbad, CA).

Cell growth, MTT, colony formation, western blot analysis, Caspase-3 activation assay, RT-PCR and quantitative real-time PCR were carried out according to our previous publications [27, 62–66]. Protein expression and purification, FP competition assay, SPR, NMR, and Wnt luciferase reporter assay were carried out as previously described [27]. The primer sequences, the primary and the secondary antibodies used were from Lan et al. [27]. Live cell imaging was carried out using EVOS FL Auto Cell Imaging System (Invitrogen, Thermo Fisher Scientific) and images were cropped and processed using ImageJ (NIH).

For all cell based studies, the DMSO concentration was 0.1% except where indicated below (for CETSA).

### Computational modeling

The AutoDock4.2.6 program [67] was used for docking calculations. The three-dimensional structure of Musashi1’s RBD1 in complex with RNA (PDB: 2RS2) was used to dock the gossypolone compound at the MSI1 RBD1 - RNA interface. A grid box of size 40*44*56 Å with 0.375 Å spacing centered around residue F23 was used for docking. A total of 200 docking runs were carried out using the Lamarckian genetic algorithm. The docked conformation with lowest energy was selected as the final predicted binding mode.

### Cellular thermal shift assay (CETSA)

CETSA was carried out according to Molina et al. [68]. For Gn dose CETSA, the HCT-116 β/W cell lysates with different concentrations of Gn were incubated for 30 min and heated individually at 52 °C for 3 min (StepOnePlus™ Real-Time PCR System, Applied Biosystems/Life Technologies) followed by cooling for 3 min at 25 °C. The soluble fractions were analyzed by western blot. The concentration of DMSO in each sample is 3.3%. Musashi-1 antibody used for CETSA was anti-MSI1 (01–1041, Millipore, Billerica, MA). Western band intensities were measured using Image Studio Ver 4.0 (LI-COR Bioscience, Lincoln, NE), and normalized to α-Tubulin.

### Preparation and characterization of gossypolone-encapsulated liposomes (Gn-lip)

Gn-lip was formed using a mixture of Gn, EPC, PEG-DSPE, and cholesterol in chloroform, at a molar ratio of 30/85/6/9. The solution was dried under vacuum to form a thin film of Gn/carrier mixture, which was then dissolved in DPBS to produce Gn-encapsulated liposomes. Blank liposomes were prepared similarly without the addition of Gn. To prepare the samples for TEM image, both Gn-lip and blank liposomes were diluted in DI water, respectively. The suspensions were applied to a grid and negatively stained by 4% uranyl acetate. Images of liposomes were acquired using FEI Tecnai G2 Polara 200 kV TEM (FEI Company, OR, USA). The size distribution and zeta potential of liposomes in DI water were measured at 25̊ C using a Malvern instrument (Nano-ZS90, Malvern, UK). The size stability of Gn-lip for 3 months was investigated at 4 °C. The drug loading efficiency (DLE%) and drug loading content (DLC%) of Gn were determined using filtration method. Gn-lip solution was filtered using an ultra centrifugal filter unit (MWCO 3000 Da, Amicon®, Merck KGaA, Germany). The concentration of free drug in the filtrate was determined using a UV-vis spectrophotometer. The DLE% and DLC% of Gn were calculated as follows: DLE% = (weight of loaded Gn ÷ total weight of input Gn) × 100%; DLC% = (weight of loaded Gn ÷ total weight of Gn-lip) × 100%.

The viabilities of HCT-116 and DLD-1 cells in the presence of free Gn or Gn-lip were determined using MTT-based assay, as described previously.

### Biodistribution of DiR-loaded liposomes in tumor-bearing mice

NOD.CB17-Prkdcscid (SCID) mice were purchased from Harlan laboratory (Indianapolis, IN) and bred at the University of Kansas Animal Care Unit. The in vivo tumor-specific distribution of liposomes was studied using DiR, a near-infrared (NIR) fluorescent dye. DiR-loaded liposome was formed using a mixture of DiR, EPC, PEG-DSPE, and cholesterol in chloroform, at a molar ratio of 1/85/6/9. The solution was dried under vacuum to form a thin film of DiR/carrier mixture, which was then dissolved in DPBS to produce DiR-loaded liposomes. Two DLD-1 tumor-bearing SCID mice were used for in vivo fluorescence imaging according to our previous studies with modifications [69, 70]. Briefly, 10 nmol DiR-loaded liposome in 200 μL was intravenously (*i.v.*) injected into one mouse; 200 μL 10 nmol DiR ethanol/water (1:4 *v*/v) mixed solvent as the control was *i.v.* injected into another mouse. At different time points, the biodistributions of DiR in both mice were observed using a Carestream Molecular Imaging System (Carestream Health, Rochester, NY), with excitation at 750 nm and emission at 830 nm using an exposure time of 60 s. Mice were euthanized at 72 h post-injection by CO2 overdose and confirmed by cervical dislocation as recommended by the Panel on Euthanasia of the American Veterinary Medical Association. Organs and tumors of mice were obtained for further ex vivo fluorescence imaging. The fluorescence intensities of tumors at different time point in vivo, and tumors and livers ex vivo, were quantified using the ‘Image Math’ function of Carestream Molecular Imaging Software (Carestream Health, Inc). To produce calibration curves for DiR-lip and free DiR, 50 μL DPBS containing different amount of DiR-lip or free DiR was added in each well of a 96-well plate, followed by in vitro imaging using the same settings with that of the in vivo imaging. The calibration curves were produced using the fluorescence intensity of each well. The amount of dye in each tissue was calculated using its fluorescence intensity and the corresponding calibration curve. The fluorescence percentage of injected dose per gram (%ID/g) of each tissue was calculated using the following formula:

$$ \%\mathrm{ID}/\mathrm{g}=\frac{{\mathrm{M}}_{\mathrm{DiR}}\kern0em }{\mathrm{ID}\times {\mathrm{W}}_{\mathrm{Tissue}}}\times 100\% $$

in which M_DiR_ is the amount (nmol) of DiR in the tissue, ID is the injected amount (nmol) of DiR, and W_Tissue_ is the weight (g) of tissue.

### In vivo drug efficacy of Gn in DLD-1 tumor-bearing nude mice

The in vivo experiments were carried out with 5 to 6-week-old female athymic NCr-nu/nu nude mice purchased from the Harlan laboratory (Indianapolis, IN). After alcohol preparation of the skin, mice were inoculated subcutaneously with 200 μL DLD-1 cell suspension (1 × 10^6^ cells) in plain DMEM on both flanks using a sterile 23-gauge needle. When tumors reached 40 mm^3^ on average, the mice were randomized into 2 groups. Group 1 (10 mice, 20 tumors) was given vehicle as the control; group 2 (5 mice, 10 tumors) was given 10 mg/kg Gn-lip. Gn-lip was administrated intravenously 2 times weekly for 3.5 weeks. Tumor size and body weight of each mouse were measured twice a week, and tumor volumes were determined as *a* × *b*^2^/2, in which *a* and *b* represent the longest and shortest diameter of the tumors, respectively. All animal experiments were carried out according to the protocol approved by the Institutional Committee for the Use and Care of Animals of University of Kansas.

### Statistical analysis

Using Prism 5.0 software (GraphPad Prism), one-way ANOVA and *t*-Test were used to analyze the in vitro data, two-way ANOVA was used to analyze the in vivo data. A threshold of *P* < 0.05 was defined as statistically significant.

## Results

### Gossypolone disrupts the Musashi-numb RNA interaction

**Fig. 1:**
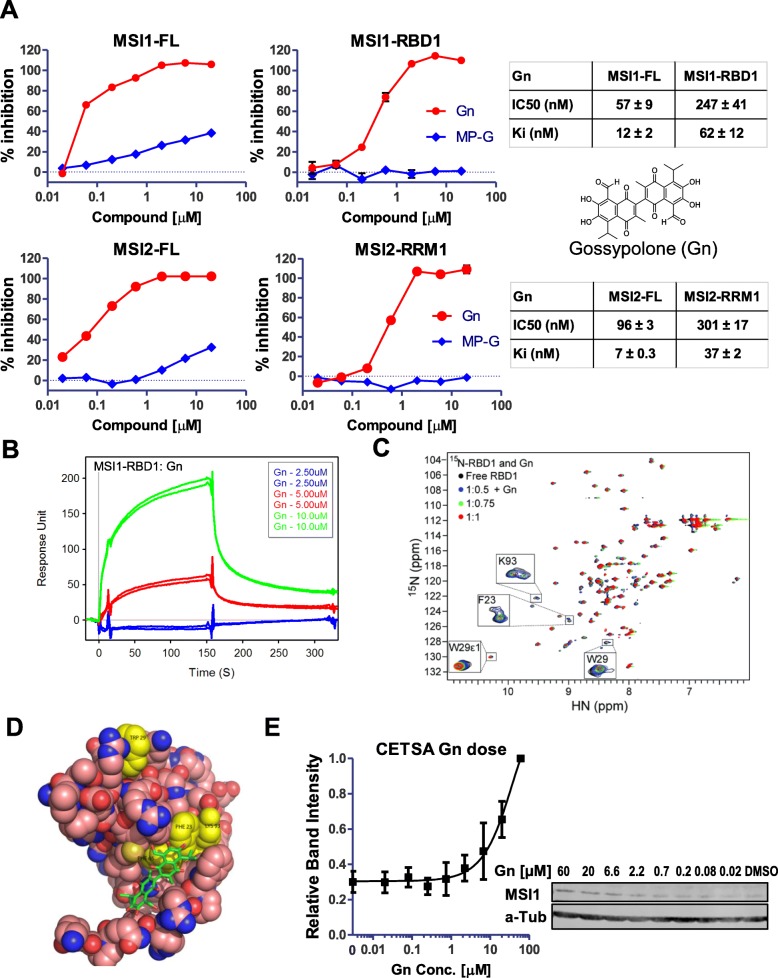
Gossypolone disrupts Musashi-*numb* RNA binding and directly binds to RBD1 of MSI1. **a** Gossypolone (Gn) was identified as a potential MSI1 inhibitor through an initial FP-based drug screening. Top panel: Dose-response curves of Gn and its inactive analog MP-Gr in Full length MSI1 (MSI1-FL) or RNA Binding Domain 1 (aa 20–107) of MSI1 (MSI1-RBD1) to *Numb* RNA. Bottom panel: Dose-response curves of Gn and MP-Gr in Full length MSI2 (MSI2-FL) or RNA Recognition Motif 1 (aa 20–107) of MSI2 (MSI2-RRM1) to *Numb* RNA. Ki values were calculated based on the Kd and the dose-response curves. **b** SPR analyses of Gn binding to immobilized GB1-tagged MSI1-RBD1. Higher response unit (RU) is a result of more binding events. **c** Overlay of 2D ^1^H-^15^N HSQC spectra of ^15^N-MSI1-RBD1 (black) titrated with Gn. Four RNA binding residues (boxed) undergo line broadening upon addition of Gn indicating that they are involved in binding to Gn. **d** Docked model of MSI1 RBD1 bound to gossypolone. The protein atoms are shown as spheres. The gossypolone structure is shown as sticks. The four MSI1-RBD1 RNA binding residues that undergo significant shifts are highlighted in yellow (F23, W29, F65 and K93). This figure was prepared using PyMOL. **e** Gn dose-response CETSA in HCT-116 β/W cell lysate (*n* = 2) with one representative western blot

In our previous screen for small molecule inhibitors of MSI1-*Numb* RNA binding using FP competition assay, we identified and validated (−)-gossypol as an effective inhibitor that disrupts MSI1-RNA binding [27]. We also identified gossypolone (Gn) as a potent disruptor of MSI1-*Numb* RNA binding, with more than 20-fold higher affinity than that of (−)-gossypol under the same experimental condition (Ki 13 ± 5 nM vs 476 ± 273 nM) [27]. Figure 1a showed that Gn dose dependently inhibits MSI1 from binding to a fluorescein labeled *Numb* RNA (5’-UAGGUAGUAGUUUUA-3′), with Ki of 12 nM and 62 nM to full length MSI1 (MSI1-FL) and RNA-Binding Domain 1 (RBD1) of MSI1 (MSI1-RBD1) respectively. As a control, MP-Gr, which is structurally related to Gn and (−)-gossypol, showed a Ki of larger than 200 μM (Fig. 1a top panel, [27]). Because of the conserved residues in N-terminal RRMs of MSI1 and MSI2 [43], the same *Numb* RNA in our MSI1 FP assay also binds to MSI2-FL and MSI2-RRM1 (data not shown). Figure 1a bottom panel showed that Gn disrupted MSI2-*Numb* RNA binding, with Ki of 7 nM and 37 nM to full length MSI2 and MSI2-RRM1 respectively. Our data demonstrate that Gn disrupts MSI1/MSI2-*Numb* RNA binding and can potentially work as a MSI1/MSI2 dual inhibitor.

### Gossypolone directly binds to the RBD1 of MSI1 protein

To confirm the direct binding of Gn to MSI1, we carried out additional assays. First, we tested Gn in a SPR-based binding assay. In SPR, GB1-tagged MSI1-RBD1 was immobilized on the sensor chip and the level of response increases with increasing amount of material bound to the surface. As shown in Fig. 1b, at 5 μM, the response was 50, while at 10 μM, the response was 200. The SPR assay showed that Gn binds to MSI1-RBD1 in a dose-dependent manner.

The binding of Gn to MSI1-RBD1 was also confirmed using NMR (Fig. 1c). Results of NMR titrations of ^15^N MSI1-RBD1 with Gn showed that the RNA-binding residues (K93, F23, and W29) were primarily affected by Gn (Fig. 1c). The backbone amide peaks of K93, F23, and W29; including the side chain peak of W29, showed changes in peak positions as well as decreased peak intensities with increasing amounts of Gn, whereas most non-RNA binding residues remained unaffected. These NMR results suggested that these residues are involved in the binding of Gn to the RNA-binding pocket of MSI1-RBD1. Using computational docking, we then built a model of Gn bound to the RNA-binding pocket of MSI1-RBD1 (Fig. 1d); this model is in agreement with the NMR observation that these particular residues (K93, F23 and W29) are responsible for the interaction of MSI1-RBD1 with Gn.

### Gn targets MSI1 in cells

To test drug-target engagement in cells, we used the CETSA to determine the thermal stability of target protein MSI1. When a protein is stabilized with addition of a ligand, the bound proteins can stay in solution whereas unbound proteins denature and precipitate with increasing temperatures [68]. The advantage of CETSA is that one can evaluate the compounds in a cellular context, thus allowing us to identify the compounds with poor bioavailability that otherwise have high affinity in biochemical assays. Fig. 1e showed the concentration-dependent target engagement of Gn with MSI1, that is, more MSI1 protein is stabilized at higher Gn concentration.

### Gn inhibits cell proliferation, induces apoptosis and autophagy in colon cancer cell lines

**Fig. 2:**
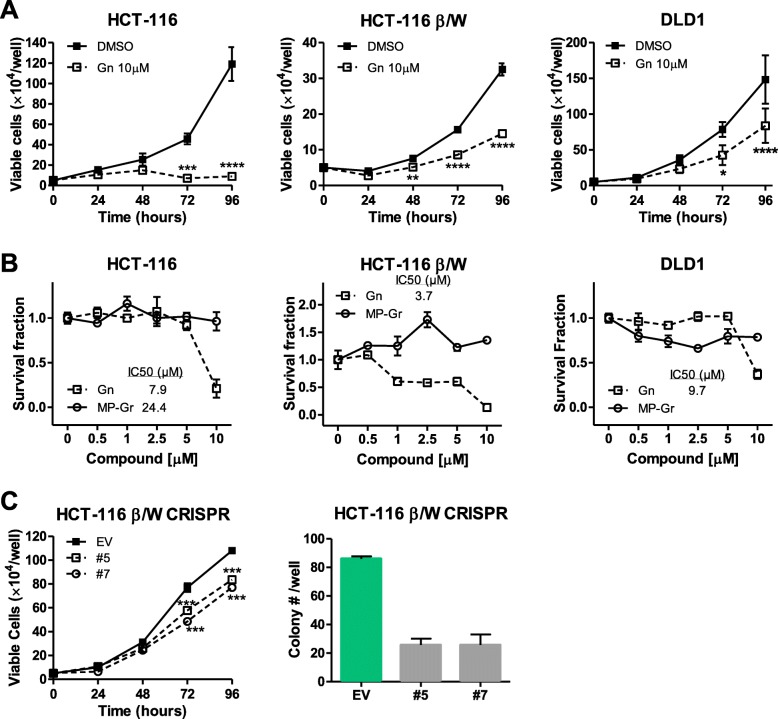
Gn inhibits cell proliferation in colon cancer cell lines. **a** Gn inhibits HCT-116, HCT-116 β/W and DLD-1 cell growth (*n* = 3). **b** Colony formation assay with different doses of Gn and MP-Gr (n = 3) in HCT-116, HCT-116 β/W and DLD-1 cells. **c** Cell growth assay and colony formation assay in HCT-116 β/W MSI1 CRISPR knockout clones. In all figures, ****p < 0.0001, ***p < 0.001, **p < 0.01, *p < 0.05 versus DMSO control. MP-Gr or DMSO treatment is consistent with our early report [27]

Previous studies have pointed to a tumorigenic role for MSI1, with overexpression of MSI1 leading to tumorigenesis in a mouse xenograft model [71], and decreased MSI1 leading to reduced tumor progression [3, 4, 10]. Our in vitro biophysical binding studies revealed a role of Gn in disrupting the RNA-binding ability of MSI1. We hypothesized that such disruption would lead to a de-repression of MSI1 target mRNA translation, thus decreased Notch/Wnt signaling and decreased cell growth. To investigate the effect of Gn in cells, we first assayed the overall growth of colon cancer cells with Gn treatment. As shown in Fig. 2, compared to negative controls DMSO or MP-Gr [27], 10 μM Gn treatment led to a significant decrease in cell growth in three colon cancer cell lines tested (Fig. 2a), colony formation assays also confirmed that there were fewer colonies formed with higher concentrations of Gn (Fig. 2b). Gn treatment phenocopied the cell growth assay and colony formation assay results obtained with HCT-116 β/W MSI1 CRISPR knockout clones (Fig. 2c, Additional file 1: Figure S1) and HCT-116 β/W MSI1 shRNA knock down clones (data not shown).

**Fig. 3:**
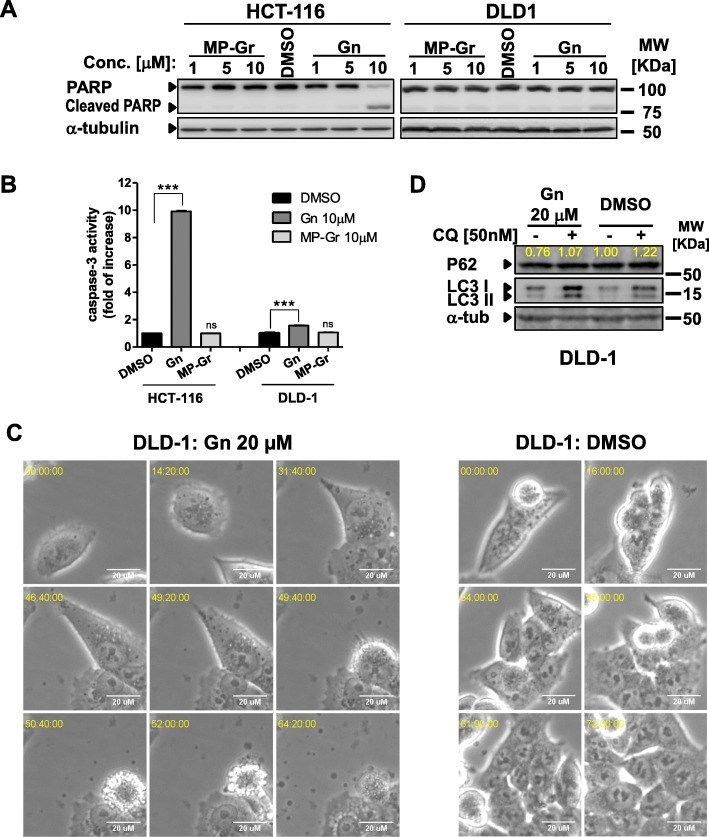
Gn induces apoptosis and autophagy in colon cancer cell lines. **a** PARP cleavage was observed in colon cancer cell lines treated with different doses of Gn for 48 h. MP-Gr or DMSO treatment had no effect, consistent with our early report [27]. **b** Caspase-3 activity was increased in Gn treated cells. MP-Gr or DMSO treatment had no effect, consistent with our early report [27]. (n = 2). ***p < 0.001 versus DMSO control. **c** Representative images of Gn or DMSO treated DLD-1 cells from time lapse videos. **d** DLD-1 cells were treated with Gn or DMSO, in the presence or absence of chloroquine (CQ, 50 nM) pretreatment for 16 h


We next tested whether Gn treatment will induce apoptosis and/or autophagy in cells. We examined PARP cleavage and Caspase-3 activation in two colon cancer cell lines. As shown in Fig. 3a-b, at 10 μM, Gn led to increased PARP cleavage (Fig. 3a), as well as augmented Caspase-3 activation (Fig. 3b); while MP-Gr or DMSO did not show any effect, consistent with our early report [27]. These data indicate that Gn induces apoptosis in colon cancer cell lines. Using a live cell image system, we showed that 20 μM Gn induced autophagy in DLD-1 cells and led to cell death via apoptosis (Additional file 2: Video 1 and Additional file 3: Video 2). One representative view from the video in each treatment was presented in Fig. 3c. The cell treated with 20 μM Gn started to accumulate autophagosome after 14 h, and died via apoptotic cell death (Fig. 3c left panel). In contrast, cells with DMSO control proliferated and covered the whole view at the end of the time lapse (72 h) (Fig. 3c right panel). Additionally, autophagy induction was shown by the LC3 conversion and P62 degradation [72] in Gn treated samples (Fig. 3d). When we pretreated the cells with chloroquine (CQ), an autophagy inhibitor that blocks the fusion of autophagosome with lysosome and lysosomal protein degradation [73], p62 degradation was blocked (Fig. 3d). These data indicate that Gn induces efficient autophagic flux, and leads to apoptotic cell death.

**Additional file 2: Video 1.** (AVI 2064 kb)



**Additional file 3: Video 2.** (AVI 2645 kb)


### Gn down-regulates Notch/Wnt signaling through MSI1

**Fig. 4:**
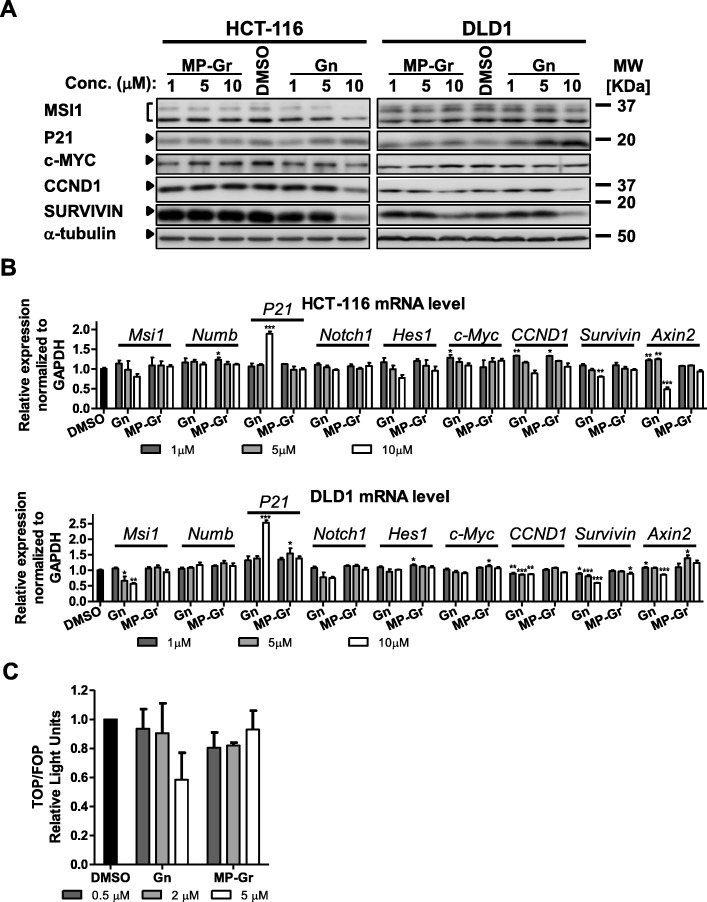
Gn down-regulates Notch/Wnt signaling. In HCT-116 and DLD1 cells, Notch and Wnt target genes expression in protein (**a**) and mRNA (**b**) levels were altered upon drug treatment. For protein detection, cells were collected 48 h after drug treatment; for real-time RCR, cells were collected 24 h after treatment. **c** TOP flash Wnt signaling reporter assay was carried out in HCT-116 cells with DMSO or different doses of drugs. In all Figures, ****p < 0.0001, ***p < 0.001, **p < 0.01, *p < 0.05 versus DMSO control. MP-Gr or DMSO treatment is consistent with our early report [27]

As describe above, binding assays showed that Gn bound to RBD1 of MSI1 and potentially blocked MSI1-target mRNAs binding, which would presumably lead to changes in MSI1 downstream targets. To test this idea, we examined the levels of several proteins and mRNAs upon Gn treatment. We noticed an increase in P21 protein level with Gn treatment in both colon cancer cell lines tested (Fig. 4a). P21 is a direct binding target of MSI1 [21], Gn binding to MSI1 would release P21 from its translation repression. However, we saw an increase in P21 mRNA level as well (Fig. 4b), which may potentially be due to other effects of Gn not related to MSI. Gn is an active metabolite of (−)-gossypol, and we and others previously reported (−)-gossypol as a Bcl-2 inhibitor [62, 63, 74–76]. The increase in P21 mRNA level could be due to Bcl-2 related functions of Gn. With Gn treatment, we observed decreases in other MSI1 downstream targets as well. These included c-MYC, CCND1 (CYCLIN D1) and BIRC5 (SURVIVIN), all of which are downstream of Notch/Wnt pathways. Additionally, we detected decreases in MSI1 protein and mRNA levels, such reductions are results of decreased Wnt signaling, as MSI1 is a Wnt target [19, 71]. To evaluate the Gn’s ability in inhibiting Wnt signaling, we used a TOP/FOP reporter assay. As shown in Fig. 4c, Gn dose-dependently inhibited the reporter activity. Taken together, our data indicate that Gn down-regulates Notch/Wnt signaling.

Compare with (−)-gossypol, Gn was less effective in downregulating Notch/Wnt signaling through MSI1 in cells. For example, Wnt target gene *AXIN2* mRNA levels were 50% (10 μM Gn treatment) versus 20% (10 μM (−)-gossypol treatment) compared to DMSO control (set as 1) in HCT-116 cells, and 80% versus 40% in DLD-1 cells (Fig. 4b, [27]). In our biophysical assays, Gn showed a better affinity to MSI1 (Fig. 1a, [27]). We thus sought to introduce a carrier for delivering Gn in vivo.

### Characterizations of Gn-lip

**Fig. 5:**
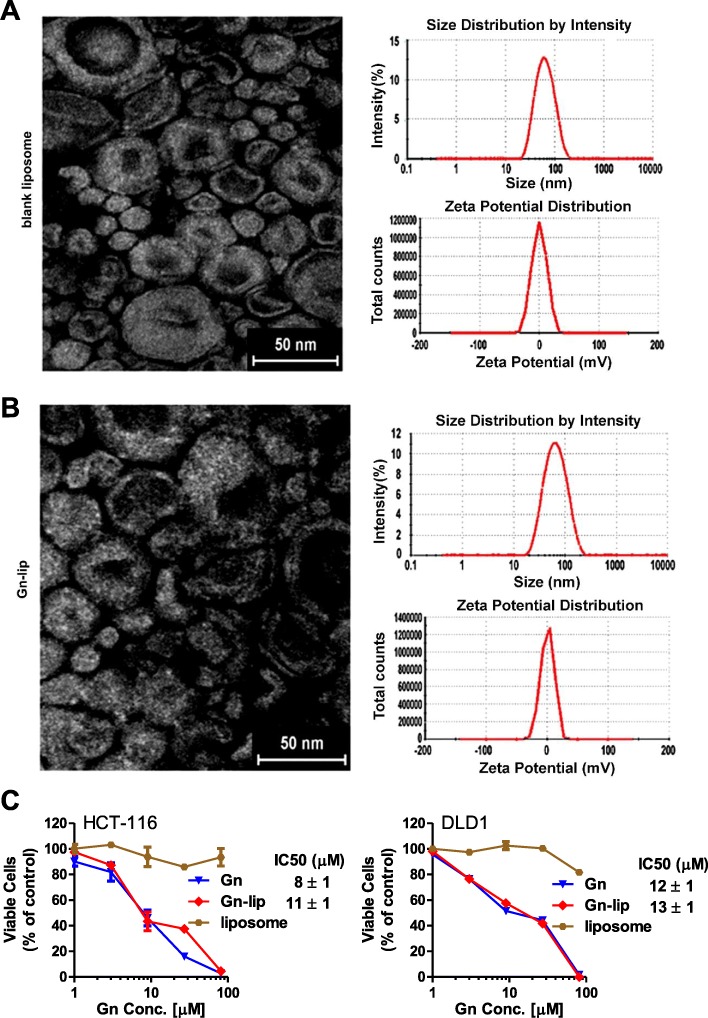
Characterization of gossypolone-liposomes (Gn-lip). **a** TEM image (left), size distribution (upper right), and Zeta-potential (lower right) of blank liposomes. **b** TEM image (left), size distribution (upper right), and Zeta-potential (lower right) of Gn-loaded liposomes. **c** MTT-based cytotoxicity assay of free Gn, encapsulated Gn (Gn-lip), and the liposomes with the same concentrations of vehicle in Gn-lip using selected colon cancer cell lines (n = 3). Gn-lip and Gn showed similar cell viability profiles; while the liposomes alone did not show evident cytotoxicity within the investigated concentrations

The morphology of Gn-loaded liposomes (Gn-lip) and blank liposomes was observed using transmission electron microscopy (TEM). Both Gn-lip and blank liposomes exhibited a similar spherical shape (Fig. 5a and b on the left). Some shrinkage was also observed in larger liposomes. No obvious difference was found between the two samples. Also, both liposomes possessed similar dynamic sizes around 56 nm (55.89 ± 0.34 nm for Gn-lip and 56.03 ± 0.42 nm for blank liposomes) and Zeta-potential around zero mV (− 0.04 ± 0.06 mV for Gn-lip and 0.83 ± 0.46 mV for blank liposomes) as determined by Dynamic Light Scattering (DLS). The morphology and surface charge of liposomes were not affected by the Gn encapsulation. The DLE% and DLC% of Gn were 80.74% ± 0.77 and 13.22% ± 0.11%, respectively, respectively. After storage at 4 °C for 3 months, the particle size of Gn-lip was 62.97 ± 1.65 nm, which was near the particle size (55.89 ± 0.34 nm) of fresh sample. This result demonstrated a good size stability of Gn-lip.

The viabilities of cells in the presence of free Gn or Gn-lip were similar, for both HCT-116 and DLD-1 cells (Fig. 5c). The IC_50_ value of free Gn and Gn-lip were 12 μM and 13 μM for DLD-1 cell, respectively, and were 8 μM and 11 μM for HCT-116 cell, respectively. Although the IC_50_ values of Gn-lip was a little higher than that of free Gn, cytotoxicity of Gn was not significantly compromised by encapsulation. The increased IC_50_ values of Gn-lip might be due to the sustained release of Gn from the liposomes.

**Fig. 6:**
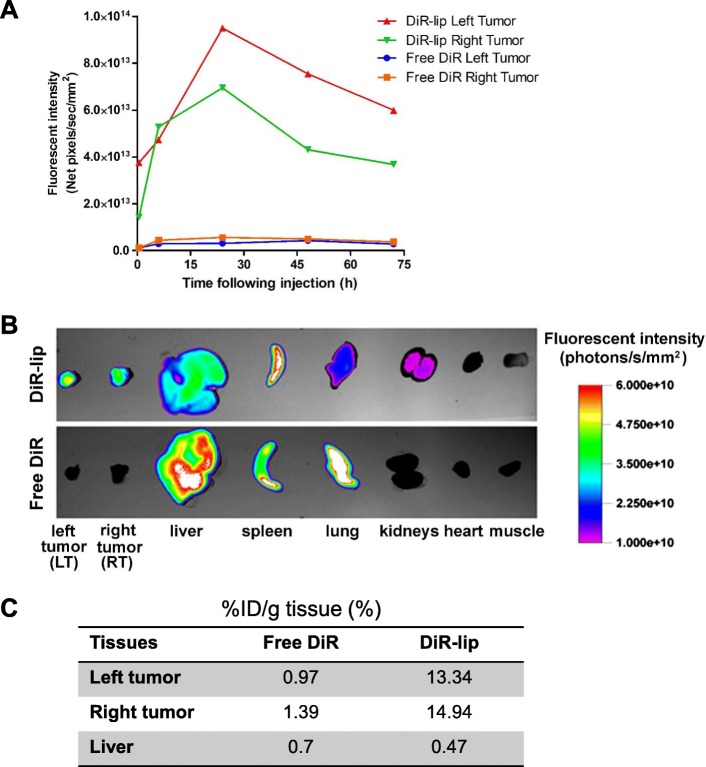
NIR imaging and biodistribution of DiR-loaded liposomes (DiR-lip) in SCID mouse bearing DLD-1 tumor. **a** In vivo DiR fluorescent intensity in tumors of mice. **b** Ex vivo NIR images of tumors and different organs of each mouse. **c** %ID/g tissue (%) for tumor and liver. Compared with control mouse, DiR in liposomes tended to accumulate in tumors rather than liver and other organs of mouse

The in vivo tumor-specific accumulation of the liposomes was confirmed using DLD-1 tumor-bearing SCID mice. In the mouse that was given NIR dye-loaded liposomes, DiR signal increased in the tumor regions over time and became the strongest 24 h after the injection (Fig. 6a). DiR signal was much weaker in control mouse that was given free DiR at the same time, due to non-specific distribution, fast clearance, and quenching of free DiR molecules. The ex vivo imaging results were shown in Fig. 6b, and the fluorescence %ID/g tissue is shown in Fig. 6c. Compared with in vivo imaging, ex vivo imaging does not have the masking effects of the skin and hairs on the fluorescence. Consistent with the in vivo results, quantifications using %ID/g also showed more DiR in tumors of DiR-lip group than in tumors of free DiR group. In addition, more DiR existed in the liver of free DiR group. Since liver is the main organ for Gn metabolism, this result indicates that the drug in liposomes may have a long-term effect as compared with the free drug. The results are also consistent with our previous findings, which indicated the elongated retention and protection of DiR in the body brought about by the encapsulation of liposomes [77].

### In vivo drug efficacy of Gn in DLD-1 tumor-bearing nude mice

**Fig. 7:**
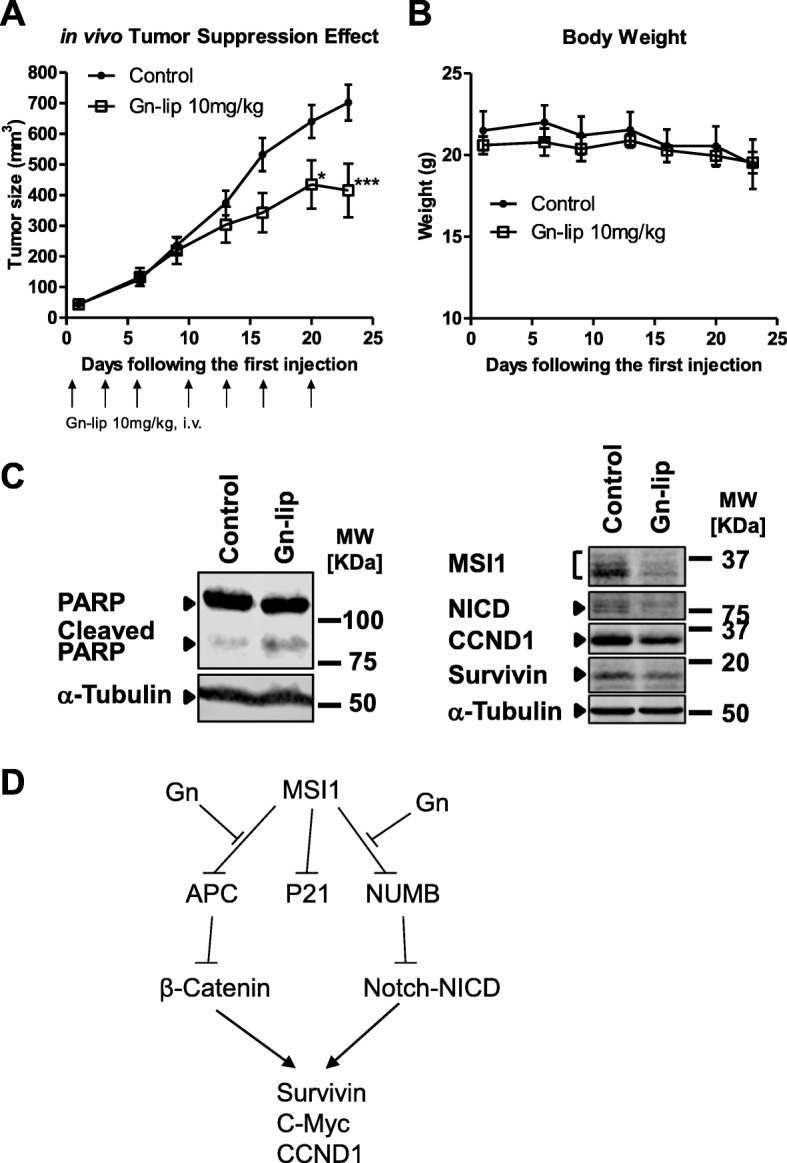
Gn-lip inhibited DLD-1 xenograft growth. Tail vein injection of Gn-lip inhibited DLD1 xenograft growth and was associated with decreased Notch/Wnt signaling and increased apoptosis. **a** Gn potently inhibited the DLD-1 xenograft tumor growth in nude mice as a single-agent therapy. The data shown are average tumor size (means ± S.E.M., *n* = 10) versus control (*n* = 20). ** *p* < 0.01 **b** Body weight of both groups remained similar during the treatment time, indicating a low systemic toxicity of Gn-lip. **c** Gn-lip induced apoptosis and down-regulated several Notch/Wnt downstream genes in tumor samples. **d** Working model

The in vivo tumor suppression effect of Gn-lip was compared with vehicle control (Fig. 7a). Significant tumor growth inhibition was observed in Gn-lip group compared with the vehicle (*P* < 0.01). Gn-lip also showed better efficacy compared to gossypol treated group (Additional file 1: Figure S2). The mice body weight of Gn group kept stable during the whole experimental time (Fig. 7b), indicating the low systemic toxicity of Gn-lip treatment. To investigate whether Gn induces apoptosis and inhibit Notch/Wnt signaling in tumors, tumor samples were collected and processed for western blotting analysis. With Gn-lip treatment, there was an increase in PARP cleavage, indication of apoptosis (Fig. 7c, left panel). We also probed the Gn-lip treatment group for several Notch/Wnt downstream targets, as shown in Fig. 7c right panel, Gn-lip treatment led to decreases in MSI1, activated Notch, CYCLIN D1 and SURVIVIN protein levels, indication of decreased Notch/Wnt signaling in the tumor tissues. Overall, Gn loaded in the liposomes had a significant tumor inhibition efficacy and a good biocompatibility; it also induced apoptosis and inhibited Notch/Wnt signaling in tumors.

## Discussion

In this study, we sought to identify additional small molecule inhibitors of Musashi-1(MSI1). Such inhibitors can downregulate Notch/Wnt signaling and block cell cycle progression through MSI1. To this end, we used FP-based screening and identified Gn as a potential MSI1 inhibitor. We further confirmed Gn binding to MSI1 using SPR and using NMR, identified the amino acids in the RNA-Recognition Motif 1 that were involved in the binding. Using colon cancer cell lines, we showed that Gn inhibited cell growth, induced autophagy, inhibited Notch/Wnt signaling in these cells and led to apoptotic cell death. However, compared to 10 μM (−)-gossypol [27], the same concentration of Gn was less active in cell assays. This result could be due to poor water-solubility of Gn. Therefore, we used a liposomal carrier to deliver Gn in animals. The liposome could efficiently increase the apparent solubility of Gn in water. We showed that Gn loaded liposomes induced apoptosis, inhibited tumor growth and Notch/Wnt signaling in DLD-1 xenograft model. Our study identified a new target for Gn and provided a new delivery method for this poorly bioavailable compound.

MSI1 is an RNA-binding protein that promotes cell proliferation and survival through Notch/Wnt signaling (Fig. 7d). Inhibiting MSI1 is a promising therapeutic strategy for preventing cancer cell proliferation and progression. Here we identified Gn as a more potent inhibitor of MSI1 compared to (−)-gossypol [27] in our binding assays. Our NMR studies showed that Gn bound to the same residues as the cognate RNA. The NMR peaks of the RNA-binding residues (K93, F23 and W29) showed the most changes upon titration of Gn (Fig. 1c). This suggests that these residues are involved in the binding to Gn, consistent with our docked model. The majority of non-RNA binding residues were unaffected when titrated with Gn, suggesting that the interaction between Gn and MSI1-RBD1 tends to be more localized near the RNA binding pocket as compared to the binding event between (−)-gossypol and MSI1-RBD1 [27]. This might explain the improved potency of Gn compared to (−)-gossypol. Additional X-ray crystallography studies will be helpful in determining the high resolution structure of the complex between MSI1-RBD1 and Gn.

Based on the sequence identity of RBDs/RRMs of MSI1 and MSI2, we tested the binding of Gn towards MSI2 and showed that Gn can disrupt the binding of MSI2 to *Numb* RNA. Like MSI1, MSI2 is also a member of the Musashi family of RNA-binding protein and shares similar roles as MSI1 in stem cells [28, 29]. In colorectal cancer initiation and maintenance, a recent study demonstrated the functional redundancy between MSI1 and MSI2 [78]. Thus using a MSI1/MSI2 dual inhibitor such as Gn in patients with MSI overexpression suggests an improved therapeutic outcome.

The introduction of PEGylated liposomes improved the dispersion of Gn in aqueous environment, thus making it possible to produce injectable drug solutions, which is essential for a better bioavailability of Gn. As shown in the results of MTT assay (Fig. 5c), the encapsulation of Gn using liposomes did not compromise the cytotoxicity of Gn in vitro. Finally, the Gn-lip exhibited a significant tumor inhibition efficacy and a low systemic toxicity in the mice. Our work is important in that our study provides a proof-of-concept to develop the Gn-lip as a novel molecular therapy for colon cancer with MSI overexpression.

The limitation of our current inhibitors is that they are not specific to MSI1/MSI2, because Gn and the previously reported (−)-gossypol [27] are both Bcl-2 family inhibitors as well [46–52, 62, 63, 76, 79]. With Gn treatment, we saw apoptosis/autophagy induction via Bcl-2 and cell proliferation inhibition via Wnt and/or Notch signaling pathways. Our goal is to develop potent and specific MSI1/MSI2 inhibitors, and ultimately move these new inhibitors into clinical applications in the treatment of cancers with MSI overexpression. Towards this goal, our future efforts will focus on utilizing computer modeling and medicinal chemistry for identifying new chemical scaffolds that selectively inhibit MSI1/2.

## Conclusions

Gn was identified as a MSI1/2 duo inhibitor in this study. It disrupted binding of MSI1 to its target mRNAs by binding to the RBD1 of MSI1. Gn inhibited colon cancer cell growth, induced autophagy, down-regulated Notch/Wnt signaling and led to apoptotic cell death. The introduction of tumor-targeted liposomes significantly improved the bioavailability of Gn, meanwhile maintaining its drug efficacy. Gn-lip has promising antitumor effects and biocompatibility in vivo, warranting further study to determine its suitability for cancer treatment.

## Additional files

Additional file 1: **Figure S1.** MSI1 and NUMB protein levels in HCT-116 β/W CRISPR clone cell lines. **Figure S2.** In vivo tumor suppression effect. (PPTX 548 kb)

## Abbreviations

APC: Adenomatous polyposis coli
CETSA: Cellular thermal shift assay
CQ: Chloroquine
DLC: Drug loading content
DLE: Drug loading efficiency
DLS: Dynamic Light Scattering
FP: Fluorescence polarization
Gn: gossypolone
Gn-lip: Gn-loaded liposomes
MP-Gr: (3, 4-Dimethoxyphenyl) methanimine gossypol
NIR: Near-infrared
NMR: Nuclear magnetic resonance
RBD: RNA-Binding Domain
RRMs: RNA Recognition Motifs
SPR: Surface plasmon resonance
TEM: Transmission electron microscopy
