# Supplementary figures and images for: Natural product derivative Gossypolone inhibits Musashi family of RNA-binding proteins
Source: BMC Cancer. 2018 Aug 10;18:809. doi: 10.1186/s12885-018-4704-z (PMC6086024; doi:10.1186/s12885-018-4704-z)

## Slide 1
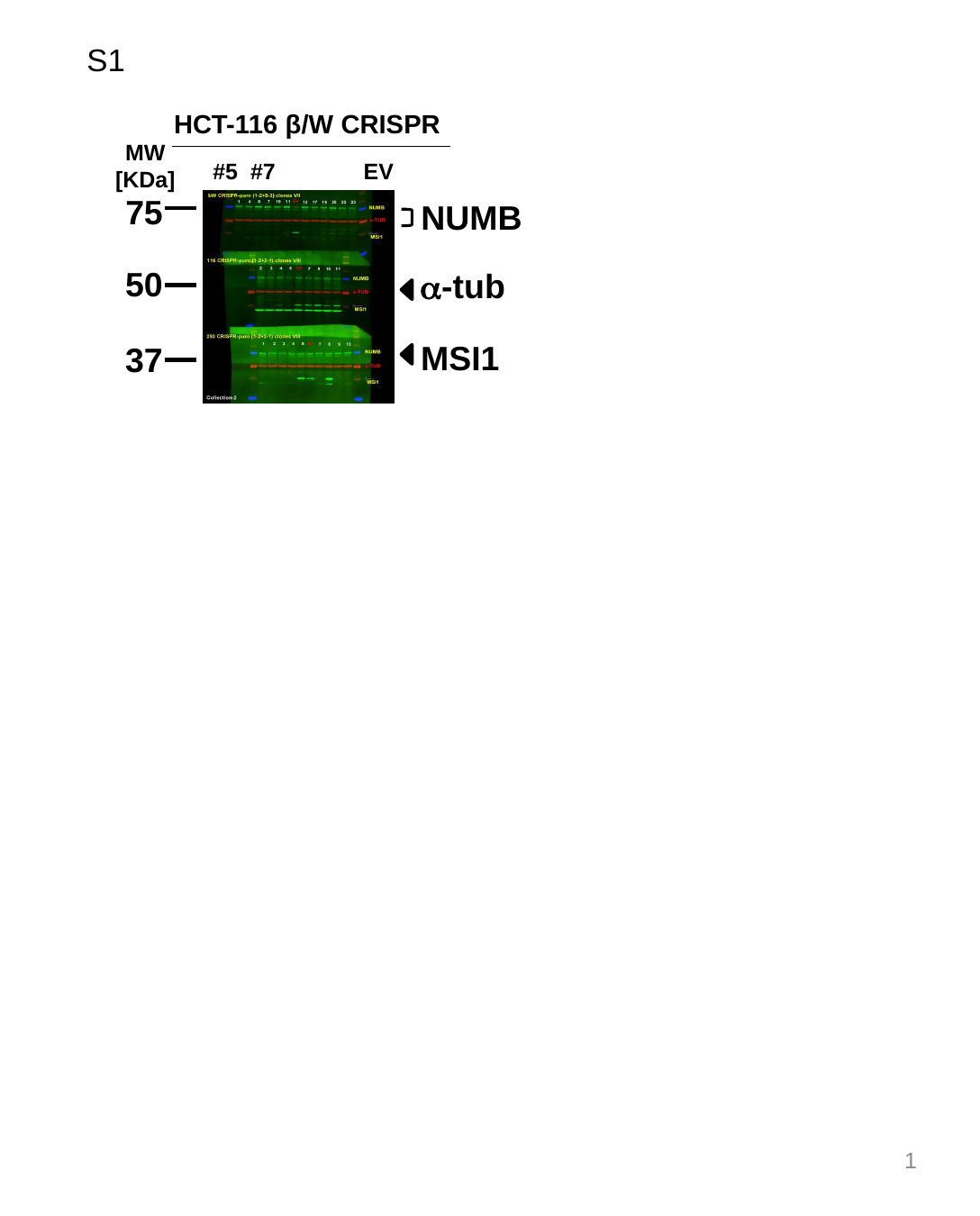

S1
 HCT-116 β/W CRISPR
#5 #7 EV
MW
[KDa]
75
NUMB
50
a-tub
MSI1
37
1

## Slide 2
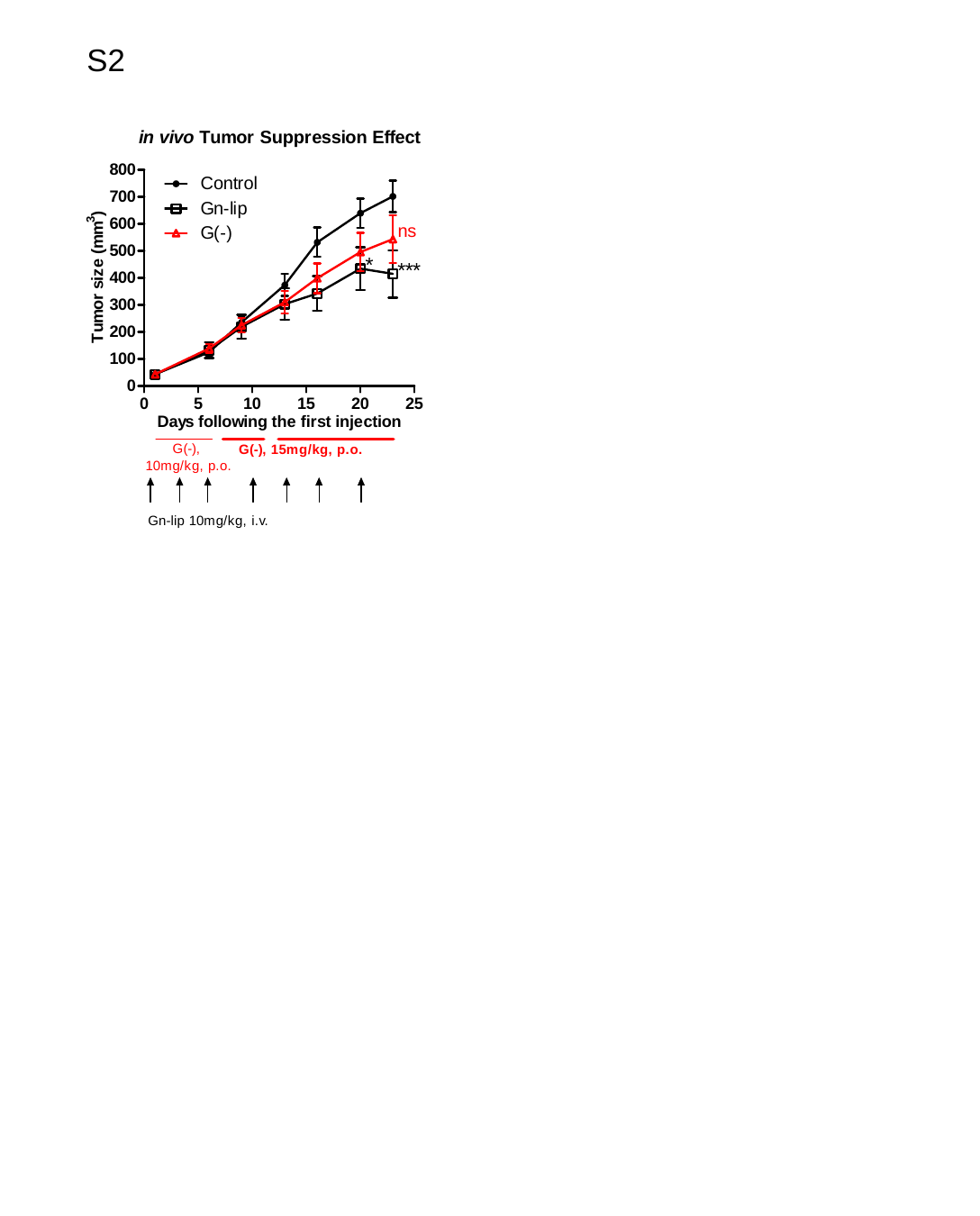

S2

Supplement: Supplementary file 3 — Figure S1. MSI1 and NUMB protein levels in HCT-116 β/W CRISPR clone cell lines. Figure S2. In vivo tumor suppression effect. (PPTX 548 kb) [file 12885_2018_4704_MOESM1_ESM.pptx]
